# F52. EFFICACY AND SAFETY OF LURASIDONE IN ADOLESCENTS WITH SCHIZOPHRENIA: INTERIM ANALYSIS OF A 24-MONTH, OPEN-LABEL EXTENSION STUDY

**DOI:** 10.1093/schbul/sby017.583

**Published:** 2018-04-01

**Authors:** Robert Goldman, Michael Tocco, Andrei Pikalov, Josephine Cucchiaro, Ling Deng, Antony Loebel

**Affiliations:** Sunovion Pharmaceuticals, Inc

## Abstract

**Background:**

Long-term efficacy and safety data from prospective studies in adolescents with schizophrenia are limited. Lurasidone is an atypical antipsychotic that has demonstrated efficacy in the treatment of schizophrenia in both adults and adolescents. The aim of the current open-label trial was to obtain long-term data on the safety and effectiveness of lurasidone in adolescents with schizophrenia.

**Methods:**

Patients ages 13–17 with schizophrenia were randomized to 6 weeks of double-blind (DB) treatment with lurasidone 40 mg/d, 80 mg/d or placebo. Patients who completed this study were eligible to enroll in a 2-year, open-label (OL), flexible-dose (20–80 mg/d) extension study in which patients were continued on lurasidone, or switched from placebo to lurasidone. These data are the results of an interim analysis of the 2-year study. Effectiveness measures included the Positive and Negative Syndrome Scale (PANSS) total score (responder criteria, ≥20% reduction from double-blind baseline).

**Results:**

A total of 271 patients completed 6 weeks of double-blind treatment and entered the 2-year extension study. At the time of the interim analysis, 132 patients had completed 52 weeks of treatment (24 patients were 2-year study completers; 96 patients were still ongoing; and 12 patients had discontinued after 52 weeks); 57 patients were still ongoing in the first 1-year of treatment; and 82 patients terminated prior to week 52 (28 patients due to withdrawal of consent; 23 due to adverse events; 9 due to lack of efficacy; and 22 for other reasons). Mean PANSS total score at double-blind baseline was 93.5. Overall mean change from double-blind to open-label baseline (after 6 weeks of treatment) was -17.5 (for patients assigned to lurasidone vs. placebo in the initial 6-week study, mean change was: -19.8 vs. -12.9). Overall mean change from double-blind baseline in the PANSS total score at weeks 28 (n=215), 52 (n=133), 76 (n=86), and 104 (n=24) was -29.2, -34.0, -35.0, and -34.1, respectively. Responder rates at week 52 and week 104 were 91.7% and 100%, respectively. During open-label treatment, the most common adverse events were headache (21.8%), nausea (11.8%), and anxiety (11.8%); 6.6% of patients reported an adverse event as severe. Median change in laboratory parameters from double-blind baseline to weeks 52 and 104, respectively, were: total cholesterol, -2.0 and -5.0 mg/dL; triglycerides, +3.5 and +3.0 mg/dL; hemoglobin A1c, 0.0 and 0.1%; prolactin in female, +0.5 and -0.5 ng/mL and males, +0.15 and +3.5 ng/mL; and mean change from DB baseline in weight at weeks 52 and 104 were 3.8 and 7.2 kg, vs. an expected weight gain of 3.3 and 5.1 kg, based on the gender-and-age specific CDC growth chart.

**Discussion:**

In adolescents with schizophrenia, long-term treatment with lurasidone was associated with continued improvement in symptoms of schizophrenia. After one year of lurasidone treatment, minimal effects were observed on body weight, lipids, and glycemic indices.

